# Supplementary material for: MetaRibo-Seq measures translation in microbiomes
Source: Nat Commun. 2020 Jun 29;11:3268. doi: 10.1038/s41467-020-17081-z (PMC7324362; doi:10.1038/s41467-020-17081-z)
Supplement: Supplementary file 10 — Supplementary Data 7 [file 41467_2020_17081_MOESM10_ESM.zip › File2/Confidence_VeryHigh_Taxonomy/442356_out.krona.html]

Javascript must be enabled to view this page.

members
magnitude
magnitudeUnassigned
count
unassigned
taxon
rank

442356\_out

17

2
superkingdom
17

1239
17
phylum

186801
17
class

186802
17
1

SRS053214\_contig\_number\_15203
order

species

SRS148511\_contig\_number\_14598
1
172733

541000
14
family

genus
13
946234

292800

SRS015264\_contig\_number\_13177SRS017521\_contig\_number\_50862SRS023346\_contig\_number\_2477SRS046712\_contig\_number\_3543SRS054956\_contig\_number\_12515SRS055966\_contig\_number\_1951SRS063127\_contig\_number\_contig-100\_14.37080SRS077730\_contig\_number\_9034SRS097889\_contig\_number\_40751SRS146764\_contig\_number\_27576SRS147022\_contig\_number\_13984SRS148511\_contig\_number\_21586SRS149244\_contig\_number\_2783
13
species

216851
1
genus

1

SRS049402\_contig\_number\_12421
species
1262898

31979
1
family

1
genus
1485

649724
species
1

SRS019601\_contig\_number\_9917
